# The usefulness of a novel sphincterotome with smooth rotatability for selective guidewire insertion

**DOI:** 10.1055/a-2427-9538

**Published:** 2024-10-25

**Authors:** Yoshihiro Goda, Kuniyasu Irie, Hiroki Sato, Hideyuki Anan, Aya Ikeda, Ryosuke Ikeda, Shin Maeda

**Affiliations:** 1 Division of Gastroenterology, Yokohama City University School of Medicine Graduate School of Medicine, Yokohama, Japan

In endoscopic ultrasound-guided hepaticogastrostomy (EUS-HGS), the fistula is a gateway for various devices, enabling procedures such as antegrade stenting, stone removal, and biopsy 1 2 3 . Selective guidewire insertion for procedures is sometimes challenging. A recently developed sphincterotome, Engetsu (Kaneka Medics, Osaka, Japan), has smooth rotatability and can undergo subtle adjustment ( Fig. 1 ). Herein, we report a case in which this novel sphincterotome was useful for selective guidewire insertion.

**Fig. 1:**
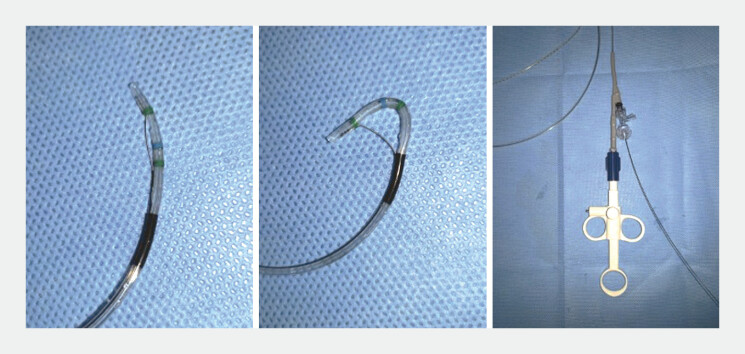
The novel sphincterotome (Engetsu; Kaneka Medics, Osaka, Japan) can undergo smooth rotation and subtle adjustment.

A 79-year-old man with failed endoscopic retrograde cholangiopancreatography (ERCP) and severe post-ERCP pancreatitis was referred to hospital because of obstructive jaundice. Computed tomography and magnetic resonance cholangiopancreatography revealed distal biliary obstruction ( Fig. 2 ). EUS-HGS was performed, and histopathological examination of the transluminal antegrade biopsy indicated adenocarcinoma 4 . To determine the extent of the cancer, an endoscopic approach using the EUS-HGS fistula was attempted with a duodenoscope (TJF-Q290V; Olympus, Tokyo, Japan).

**Fig. 2:**
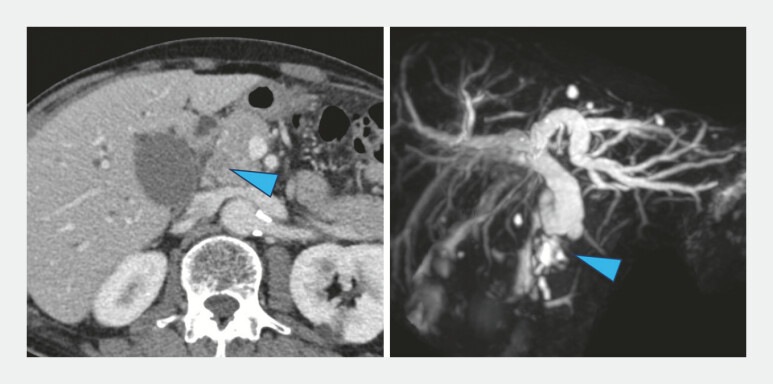
Computed tomography and magnetic resonance cholangiopancreatography showed distal biliary obstruction (blue arrowheads).

Guidewire insertion into the bile duct alongside a plastic stent with a standard ERCP catheter and a 0.025-inch guidewire was difficult because the catheter was not aligned with the plastic stent 5 . The catheter was exchanged for the Engetsu sphincterotome ( Video 1 ). By rotating the handle and bending the tip, the sphincterotome was smoothly oriented along the axis of the plastic stent, and guidewire insertion into the bile duct was successfully achieved ( Fig. 3 ). The smooth rotatability of the sphincterotome was also useful for selective guidewire insertion into the anterior branch for biopsy. With a standard ERCP catheter and a 0.025-inch guidewire, guidewire insertion was difficult because of the acute angle between the left hepatic bile duct and the anterior branch. The sphincterotome was smoothly reversed in the direction of the anterior branch by rotating the handle, enabling guidewire insertion and successful antegrade biopsy from the base of the anterior branch through an endoscopic sheath ( Fig. 4 ).

A novel sphincterotome that can undergo smooth rotation and subtle adjustment was useful for selective guidewire insertion.Video 1

**Fig. 3:**
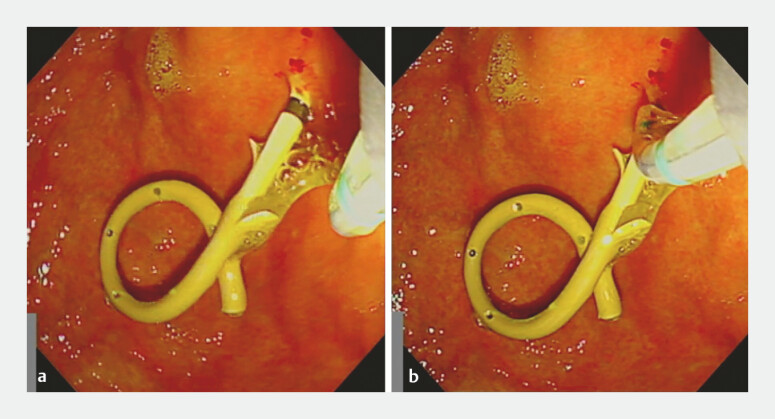
Endoscopy images. **a** Before rotation, the sphincterotome was not aligned with the plastic stent. **b** After rotation, the sphincterotome was oriented with the axis of the plastic stent.

**Fig. 4:**
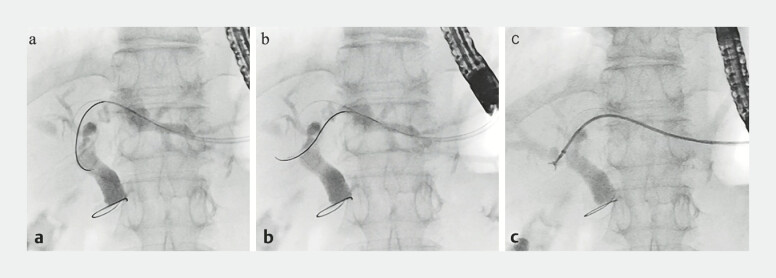
The sphincterotome was smoothly reversed in the direction of the anterior branch by rotating the handle. **a** Before rotation. **b** After rotation. **c** Successful antegrade biopsy from the base of the anterior branch.

This case highlights the smooth rotatability and subtle adjustments of this novel sphincterotome for selective guidewire insertion.

Endoscopy_UCTN_Code_TTT_1AS_2AH

## Notes

Endoscopy E-Videos https://eref.thieme.de/e-videos
*E-Videos* is an open access online section of the journal *Endoscopy* , reporting on interesting cases and new techniques in gastroenterological endoscopy. All papers include a high-quality video and are published with a Creative Commons CC-BY license. Endoscopy E-Videos qualify for HINARI discounts and waivers and eligibility is automatically checked during the submission process. We grant 100% waivers to articles whose corresponding authors are based in Group A countries and 50% waivers to those who are based in Group B countries as classified by Research4Life (see: https://www.research4life.org/access/eligibility/ ). This section has its own submission website at https://mc.manuscriptcentral.com/e-videos .
